# Case Report: Fiberoptic bronchoscopy and thoracoscopic surgery as a treatment for pulmonary mucormycosis in pediatric acute lymphoblastic leukemia

**DOI:** 10.3389/fped.2025.1591953

**Published:** 2025-06-10

**Authors:** Zhenlei Jia, Yujie Qian, Lili Zhang, Yun Li, Zhiguo Chen, Ling Zhao, Juan Du, Guangxin Tuo, Fang Yue

**Affiliations:** ^1^ Department of Thoracic Surgery, Hebei Provincial Children’s Hospital, Shijiazhuang, China; ^2^ Department of Pathology, Hebei Provincial Children’s Hospital, Shijiazhuang, China

**Keywords:** fiberoptic bronchoscopy, thoracoscopic surgery, pulmonary mucormycosis, pediatric, acute lymphoblastic leukemia

## Abstract

**Background:**

Mucormycosis, a severe disease caused by fungal infections of the Mucorales order, is a frequent cause of mortality in patients with hematological malignancies. Pulmonary mucormycosis represents a common form of this condition in pediatric patients with hematological malignancies and is associated with a high mortality rate. Liposomal amphotericin B (L-AmB) remains the primary therapeutic agent for pulmonary mucormycosis, however, its efficacy is often limited. We report a case of treating pulmonary mucormycosis in a pediatric patient with acute lymphoblastic leukemia (ALL) using fiberoptic bronchoscopy (FB) combined with thoracoscopic surgery, in conjunction with antifungal therapy including L-AmB.

**Case report and management:**

A 3-year-old male patient with a 1-year history of ALL. After the 11th chemotherapy session, the patient developed symptoms including fever, cough, and sputum production. Comprehensive diagnostic evaluations confirmed the presence of pulmonary mucormycosis in the right lung. On the basis of adequate administration of multiple antifungal agents, we conducted two fiberoptic bronchoscopy-guided bronchoalveolar lavage procedures for the child, successfully confining the lesion to the right upper lobe. However, the child continued to experience intermittent hemoptysis. Subsequently, we performed a thoracoscopic right upper lobectomy for the child. The child was discharged smoothly 9 days after the operation, and the postoperative pathology also indicated a fungal infection. One month postoperatively, a follow-up chest CT scan revealed no significant infection in either lung. Chemotherapy for ALL was continued. The child was followed up for three years after completion of treatment and remained in good health with no notable symptoms.

**Conclusion:**

The treatment of childhood ALL complicated with pulmonary mucormycosis is particularly challenging and associated with a high mortality rate. For children with severe infections, combining antifungal therapy, such as L-AmB, with bronchoscopy and thoracoscopic surgery has been shown to be both feasible and effective.

## 1 Introduction

Mucormycosis is typically classified based on the affected body systems, with five primary forms: rhino-orbital-cerebral, pulmonary, cutaneous, gastrointestinal, and disseminated (1). Pulmonary mucormycosis is an opportunistic infection caused by fungi of the Mucorales order, and it represents the second most common form of mucormycosis. This condition often presents with acute onset, rapid progression, high mortality, and generally poor prognosis (2). Hematological malignancies are a significant risk factor for developing mucormycosis (3). However, there is a relative paucity of studies focusing on the treatment of children with hematological malignancies complicated by mucormycosis. This article presents a case of a child with ALL who developed pulmonary mucormycosis during chemotherapy and was successfully treated using a combined FB and thoracoscopic approach. The treatment resulted in a favorable outcome. It is hoped that this case report will provide valuable insights into the management of children with hematological malignancies complicated by rare fungal infections.

## 2 Case presentation

### 2.1 Basic information

**Figure 1:**
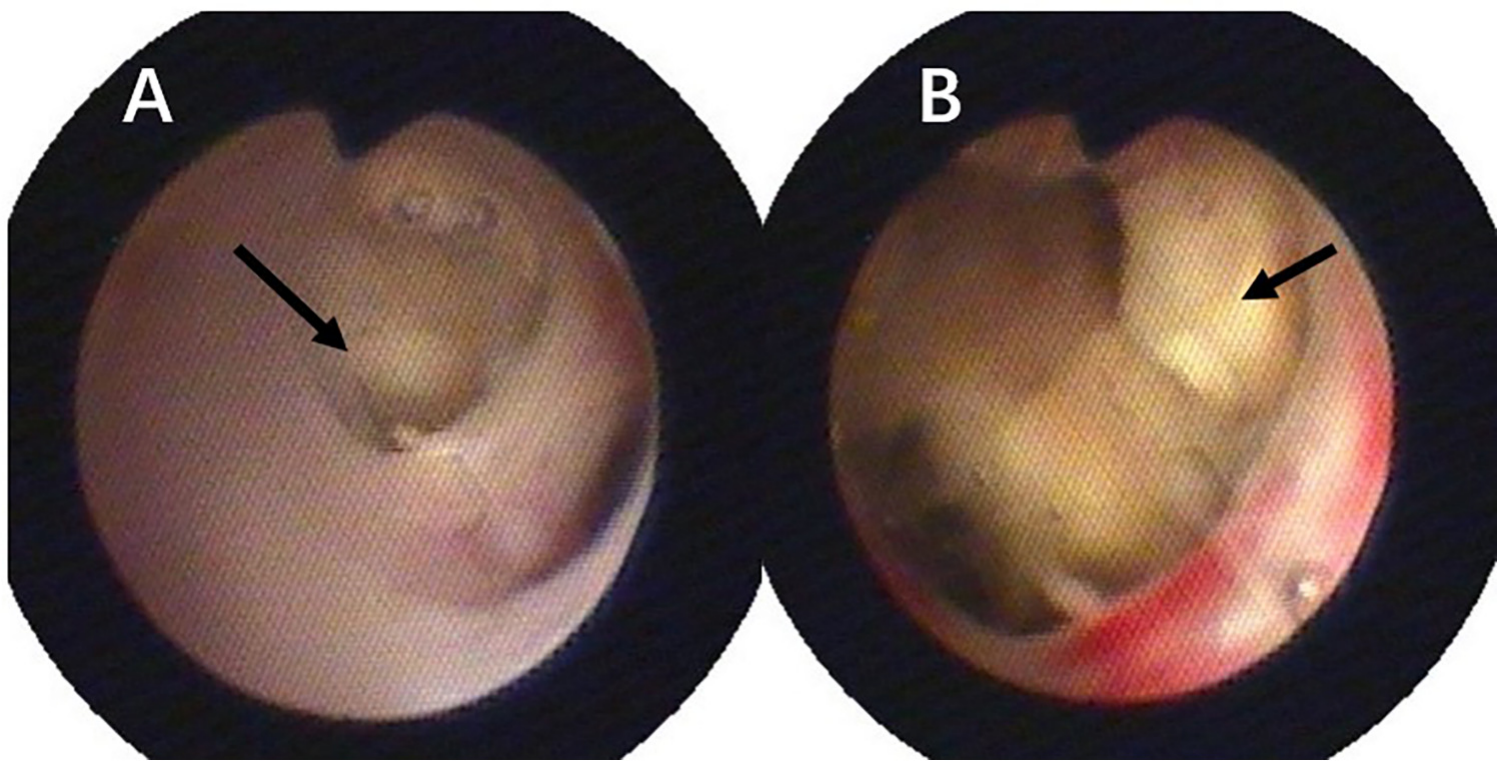
Pulmonary mucormycosis lesions observed via fiberoptic bronchoscopy, both black arrows indicate fungal masses resulting from mucormycosis: **(A)** at the orifice of the right upper lobe; **(B)** at the anterior segment of the right upper lobe.

**Figure 2:**
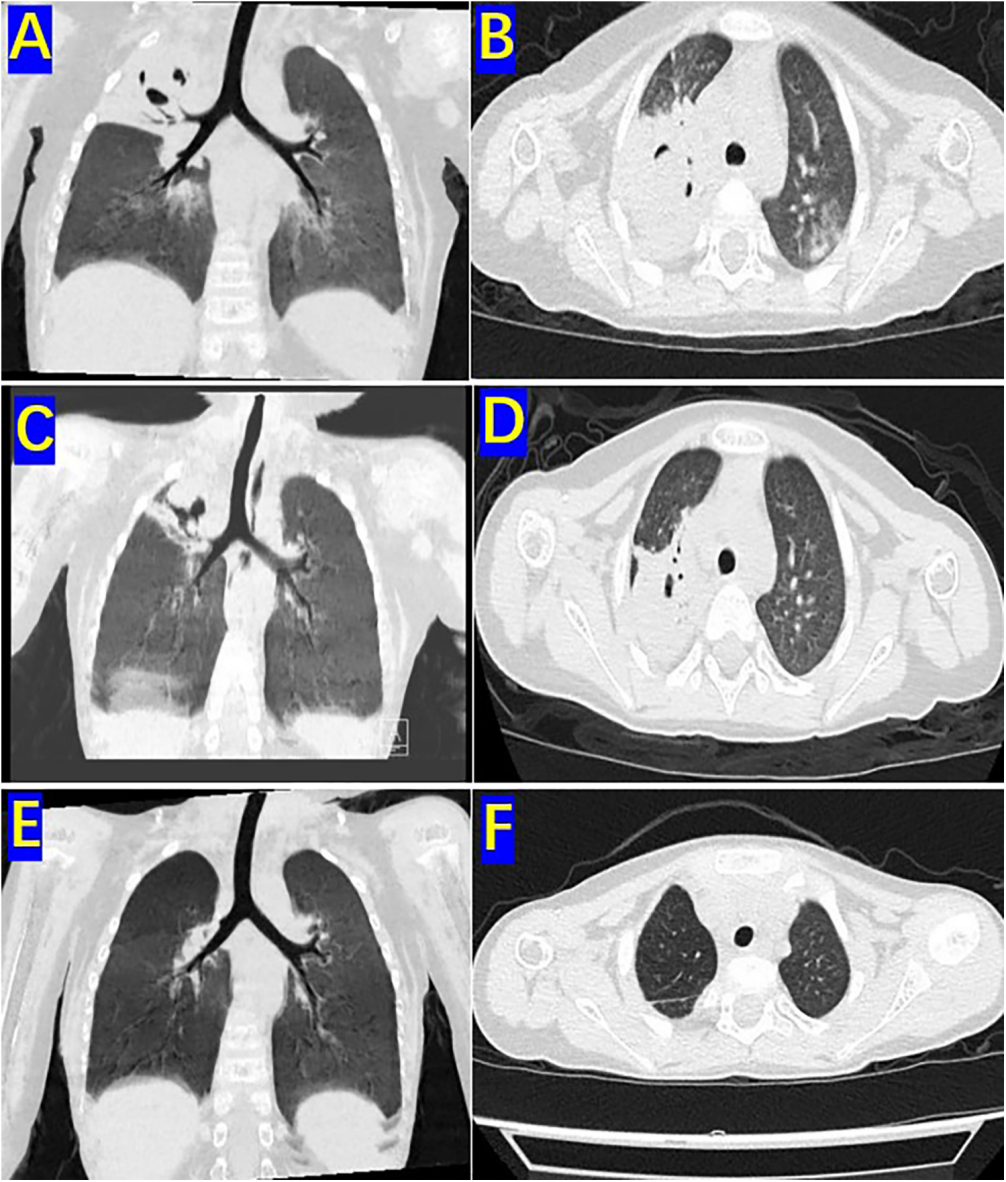
Pre-bronchoscopy treatment CT, preoperative CT and One-month postoperative CT: **(A)** sagittal view of the high-resolution chest CT lung window before two bronchoscopic bronchoalveolar lavages. **(B)** Coronal view of the right upper lobe on high-resolution chest CT lung window before two bronchoscopic bronchoalveolar lavages. **(C)** Sagittal view of the high-resolution chest CT lung window before surgery. **(D)** Coronal view of the upper lobe in the high-resolution chest CT lung window before surgery. **(E)** Sagittal view of the high-resolution chest CT lung window 1 month after surgery. **(F)** Coronal view of the upper lobe in the high-resolution chest CT lung window 1 month after surgery.

The patient was a 3-year-old boy who was diagnosed with ALL in the Department of Hematology and Oncology at our hospital one year ago. Following prednisone induction therapy, he underwent 11 cycles of chemotherapy in the same department. Two months prior to surgery, following the 11th cycle of chemotherapy, the child developed symptoms indicative of a respiratory infection, including fever, cough, and expectoration. A chest CT scan revealed extensive inflammatory lesions in the right lung, with partial consolidation predominantly in the upper lobe of the right lung (Pre-bronchoscopy treatment CT, Figures 2A,B). Initially, bacterial infection was suspected, and treatment commenced with cefoperazone-sulbactam sodium. Due to poor therapeutic response, the regimen was switched to linezolid for anti-inflammatory treatment. Subsequently, further investigations, including high-throughput gene sequencing for pathogenic microorganisms in whole blood and sputum, were performed. The patient received antifungal therapy with amphotericin B combined with oral posaconazole 1 month and 20 days prior to surgery due to infection with Rhizopus. Despite two weeks of treatment, the child continued to exhibit respiratory symptoms, including intermittent fever, cough, and expectoration. Consequently, the first FB and bronchoalveolar lavage were performed, followed by treatment with L-AmB combined with oral posaconazole. After adjusting the antifungal regimen for 2 weeks, the child's body temperature was effectively controlled. However, 20 days before surgery, the child developed hemoptysis characterized by bloody sputum, prompting a second FB and bronchoalveolar lavage. Under bronchoscopy, a fungal mass caused by Mucormycosis albuginea was observed, with complete obstruction of the anterior segment of the right upper lobe (Figure 1). Inhalation of amphotericin B was added to the original treatment regimen. After 2 weeks of treatment, the child continued to experience intermittent hemoptysis, which had slightly worsened compared to before, leading to a transfer to thoracic surgery for further management.

### 2.2 Fiberoptic bronchoscopy and bronchoalveolar lavage

Under intravenous anesthesia, lidocaine was administered for local anesthesia of the epiglottis, carina, and other relevant areas via FB. Subsequently, the FB was used to systematically examine the bronchial segments of the left lung followed by the right lung. If excessive secretions or even obstruction were detected in the bronchi, bronchoalveolar lavage was performed.

The bronchoalveolar lavage procedure involved placing the bronchoscope into the target bronchus and infusing warm normal saline (37 ℃) multiple times through the working channel of the bronchoscope (1 ml/kg per infusion, with each infusion ≤20 ml and the total volume ≤5–10 ml/kg). Following this, the bronchoalveolar lavage fluid was aspirated under negative pressure ranging from 100 to 200 mmHg (1 mmHg = 0.133 kPa), with careful selection of the negative pressure value to prevent bronchial collapse during suctioning. The recovery rate of the lavage fluid should be ≥40%. After completion of the lavage, budesonide suspension (0.5–1 mg) was injected into the target bronchus and retained.

### 2.3 Preoperative status

Two days before the operation, a follow-up chest CT scan (preoperative CT) revealed significant improvement in the inflammation of the middle and lower lobes of the right lung compared to prior findings. However, the upper lobe of the right lung exhibited extensive consolidation with cavitary changes (Figures 2C,D). The child experienced intermittent hemoptysis that progressively worsened, indicating the need for surgical intervention. After successfully correcting severe preoperative abnormalities, including hypokalemia and anemia, the patient underwent thoracoscopic surgery.

### 2.4 Thoracoscopic right upper lobectomy

After anesthesia induction, a senior anesthesiologist performed single-lung ventilation using bronchoscopy-guided bronchial blockade, achieving complete occlusion of the right bronchus. During the operation, the three-port thoracoscopic surgical approach was utilized, employing 5-mm trocars. The observation port was positioned along the midaxillary line at the 8th intercostal space, whereas the two operative ports were located along the anterior axillary line at the 5th intercostal space and along the posterior axillary line at the 6th intercostal space.

**Figure 3:**
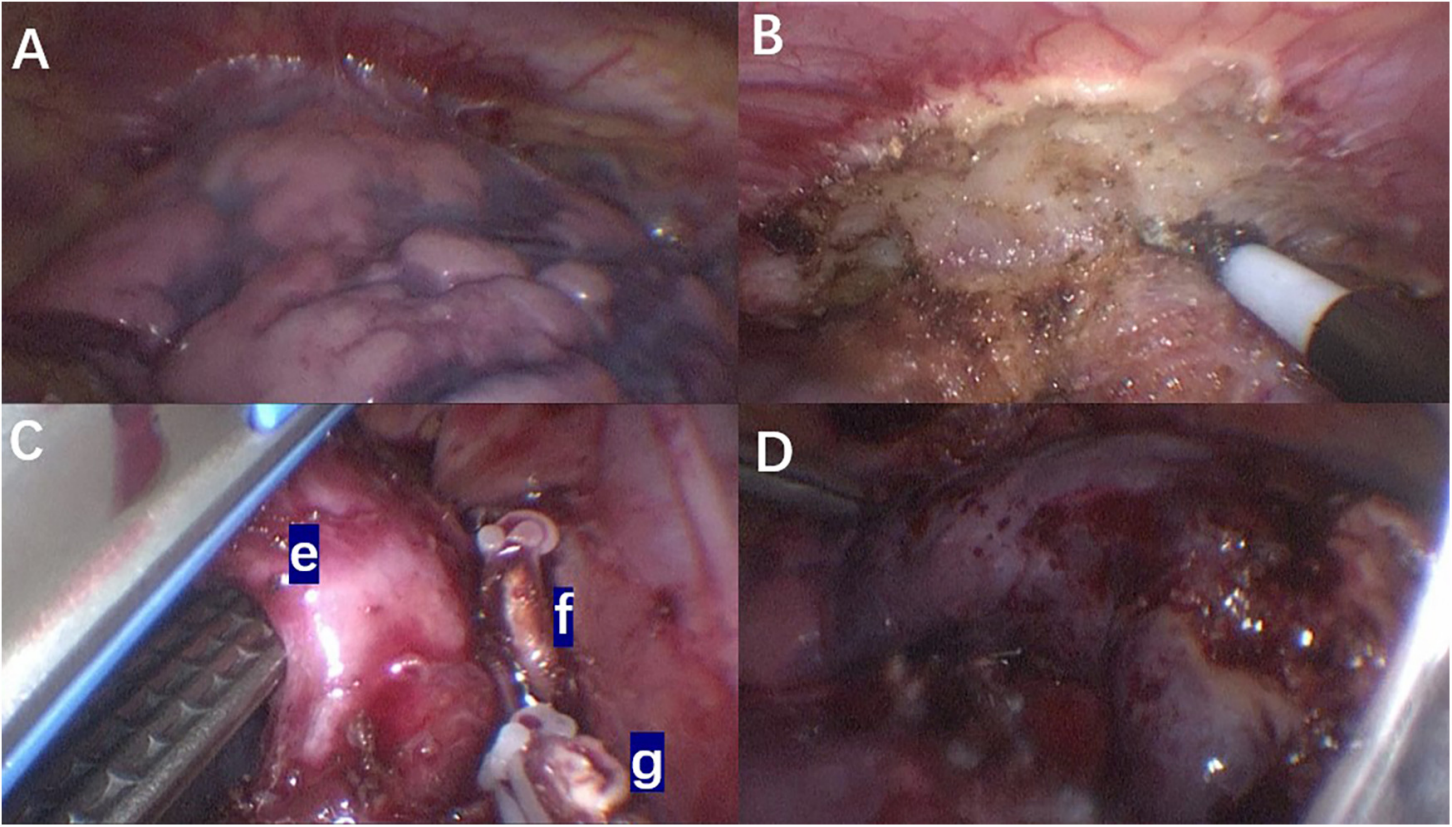
Intraoperative thoracoscopic images: **(A)** the adhesion between the upper lobe of the right lung and the chest wall; **(B)** intraoperative separation of the mucormycosis lesion from the chest wall; **(C)** during the operation, the right upper lobe pulmonary artery and vein were dissected and transected, followed by division of the right upper lobe bronchus using a linear stapler. **(e)** Right upper lobe bronchus of the lung. **(f)** Right upper lobe artery. **(g)** Right upper lobe vein. **(D)** Complete resection of the right upper lobe.

Intraoperative findings revealed that the right upper lobe of the lung was slightly reduced in volume, with its superior margin tightly adhering to the right pleural apex. The surface appeared extensively dark red, hard, and consolidated, with no ventilation function (Figure 3A). Adhesions between the upper lobe and the pleural apex were carefully separated using an electrocautery hook in combination with thoracoscopic grasping forceps (Figure 3B). The right upper lobar artery and vein were dissected free, and both proximal and distal ends were ligated with clips before being transected. Dissection proceeded from the interlobar fissure towards the hilum using an ultrasonic scalpel and thoracoscopic grasping forceps, exposing the right upper lobe bronchus. A linear cutting stapler was inserted through a 12 mm trocar, and a 30 mm staple line was used to resect the upper lobe bronchus (Figure 3C). The right upper lobe was successfully and completely resected (Figure 3D). The excised lung tissue specimen was removed. Sterile warm saline was instilled into the right thoracic cavity, and the anesthesiologist was instructed to inflate the lungs. It was observed that the lower and middle lobes of the right lung expanded well without significant air leakage. Following thorough irrigation of the thoracic cavity during surgery, a single closed thoracic drainage tube was placed to facilitate gravity drainage. The drainage status was monitored and documented daily. The color of the pleural effusion gradually transitioned from serosanguineous to pale yellow, with a progressive decrease in volume. On postoperative day 7, the 24-h drainage volume was less than 30 ml and a chest x-ray in both anteroposterior and lateral views revealed no significant fluid or air accumulation. Subsequently, the chest drainage tube was removed.

### 2.5 Postoperative management and follow-up

**Figure 4:**
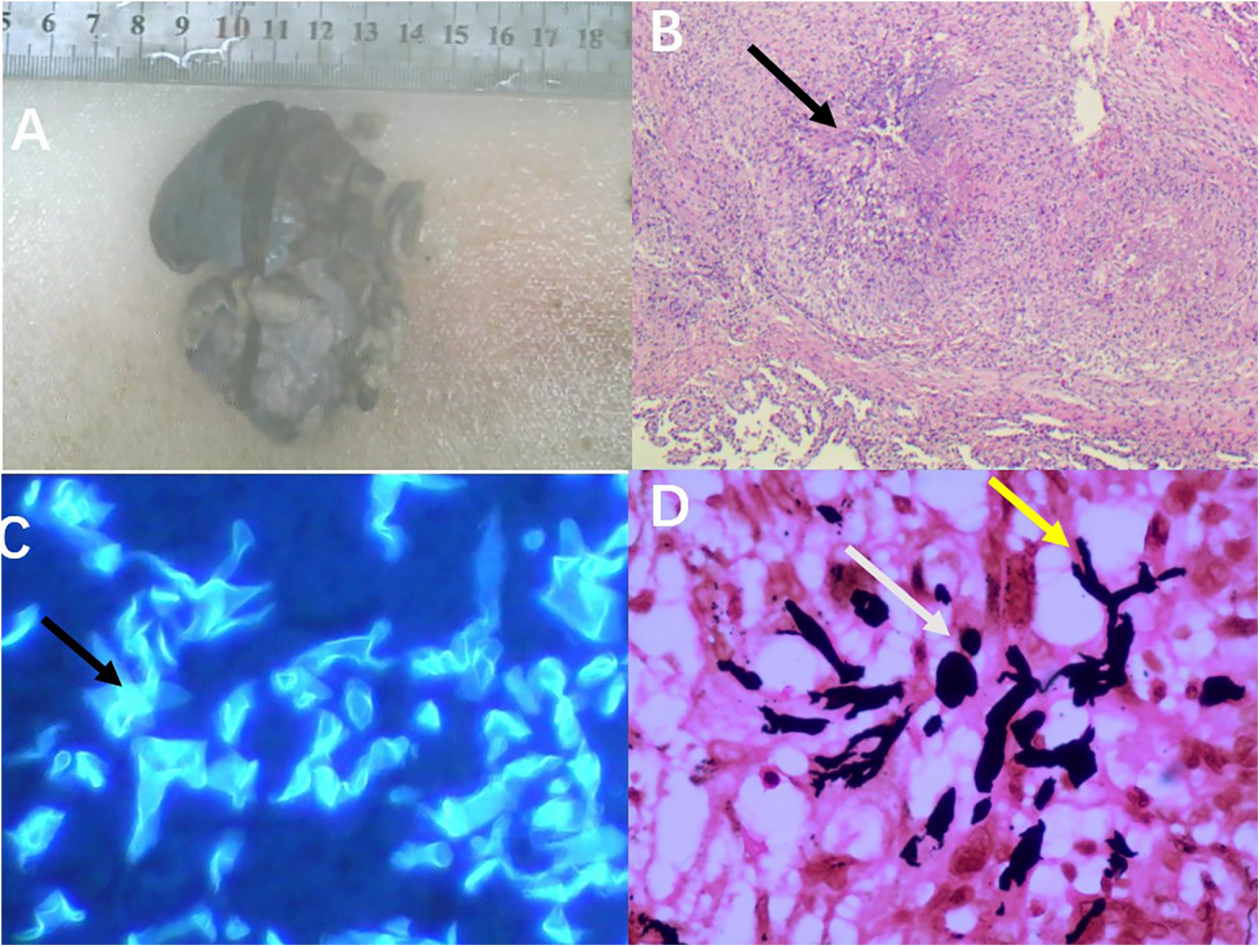
Postoperative pathological images: **(A)** the resected specimen from the right upper lobe of the lung measured approximately 7.5 cm × 5 cm × 2.5 cm. **(B)** Microscopic examination revealed necrotizing granulomatous inflammation (as indicated by the arrows, HE × 200). **(C)** Microscopic examination showed clear positive fungal fluorescence (as indicated by the arrows, 400×). **(D)** Hexamine silver staining demonstrated positivity; the yellow arrows indicate Mucor hyphae, and the white arrows indicate Mucor spores (SP × 400).

After the operation, the patient continued to receive antifungal treatment with L-AmB and was concurrently administered ceftriaxone for infection control. Additionally, our nursing team provided comprehensive postoperative recovery guidance, including nutritional support, activity planning, and pulmonary function rehabilitation exercises tailored to the child's needs. Ceftriaxone was administered for bacterial prophylaxis until postoperative day 7, when the chest drainage tube was removed and no signs of bacterial infection were observed, at which point it was discontinued. L-AmB was continued for antifungal therapy until postoperative day 9. After treatment, the patient gradually recovered and was discharged from the hospital. Following discharge, he continued oral posaconazole therapy for one month. Postoperative pathological examination revealed: (right upper lobe of the lung) necrotizing granulomatous inflammation with positive fungal fluorescence staining. Immunohistochemistry results were as follows: CK5/6 (+), TTF-1 (+), SMA (focally +), ALK (−), CD1a (−), Ki-67 (5% +). Special stains showed: PAS (−), Methenamine silver staining (+), acid-fast stain (−). PCR testing was negative for tuberculosis (TB), while fungal fluorescence was positive (Figure 4).

One month postoperatively, a follow-up chest CT scan revealed no significant signs of infection in both lungs (Figures 2E,F). Chemotherapy for ALL was continued. The child was followed up for three years after treatment completion and remained in good health with no notable discomfort.

## 3 Discussion

Over the past two decades, mucormycosis has emerged as the third most prevalent invasive fungal infection in patients with hematological malignancies, following candidiasis and aspergillosis (4). Mucormycosis is distinguished by its rapid progression, aggressive clinical course, and poor response to antifungal therapy, contributing to a high mortality rate. Studies indicate that among children with malignant tumors, the mortality rate associated with mucormycosis ranges from 41.3% to 66.6%. Notably, even in children without underlying comorbidities, the mortality rate can reach up to 16.6% (5). The predominant genera causing mucormycosis in children include Rhizopus spp. (39.7%), Lichtheimia spp. (17.5%), Mucor spp. (12.7%), Cunninghamella bertholletiae (6.3%), and unspecified species (23.8%) (5). In this case, the patient was infected with Rhizopus species.

Unlike adults, the most common sites of mold infection in children are the lungs and cutaneous soft tissues. Pulmonary mucormycosis is more prevalent in patients with hematological malignancies and neutropenia. This is because the long-term use of chemotherapy drugs, bone marrow suppression, overuse of broad-spectrum antibiotics, and central venous catheterization in patients with hematological malignancies create conditions that predispose to mucormycosis infection (6). Non-specific symptoms of pulmonary mucormycosis include fever, cough, dyspnea, and chest pain (7). Lesions typically involve the lung parenchyma but may extend to the chest wall, pulmonary artery, aorta, mediastinum, or pericardium. Pulmonary artery infiltration can lead to hemoptysis (8). In this case, the child developed mucormycosis infection following the 11th cycle of chemotherapy and subsequent bone marrow transplantation, during which the child was in a state of profound granulocytopenia. After pulmonary mucormycosis infection, the child exhibited fever, cough, expectoration, and intermittent chest pain. Following antifungal therapy and FB, the infection was partially controlled, and symptoms such as fever, cough, and expectoration improved. However, the child subsequently developed progressive hemoptysis.

Early diagnosis and treatment can significantly reduce the mortality rate of pulmonary mucormycosis (9). Prior to the introduction of metagenomic next-generation sequencing (mNGS), the diagnosis of pulmonary mucormycosis predominantly depended on fungal culture and histopathological examination, both of which have relatively low sensitivity for detecting mucormycosis (10). mNGS, a genomic-based pathogen detection method, acquires microbial nucleic acid sequence information in a single run and identifies microorganisms through bioinformatics analysis. This technique enables rapid identification of uncultivable or difficult-to-culture pathogens (11). Following the advent of mNGS, it has become crucial to promptly perform mNGS testing for children experiencing infections during the bone marrow suppression phase post-chemotherapy, especially when broad-spectrum antibiotics prove ineffective. In this case, the child received one week of broad-spectrum antibiotic therapy. Given the lack of significant improvement, blood mNGS testing was performed. Three days later, the results confirmed a diagnosis of pulmonary mucormycosis, prompting the initiation of antifungal treatment.

FB has been widely utilized in the diagnosis and treatment of various pediatric respiratory diseases (12). However, its application in pulmonary mucormycosis remains limited, with most studies being case reports. For example, one study utilized FB to diagnose a case of adult pulmonary mucormycosis and indicated that aggressive surgical intervention to debride necrotic tissue is commonly considered the primary treatment for endobronchial obstructive mucormycosis (13). Additionally, a report indicates that posaconazole monotherapy combined with bronchoscopy interventions may be a safe and effective treatment option for pediatric pulmonary mucormycosis (14). In this case, fiberoptic bronchoscopy-guided bronchoalveolar lavage was performed as an adjunct to antifungal drug therapy. Following treatment, the mucormycosis infection was confined to the right upper lobe of the lung. Subsequently, the child underwent right upper lobe resection via thoracoscopy after experiencing hemoptysis. Thoracoscopic surgery offers advantages such as reduced trauma, faster recovery, and fewer postoperative complications in pediatric lung-related surgeries, achieving comparable outcomes to open surgery (15). Studies have investigated the surgical outcomes of minimally invasive thoracoscopic procedures for pulmonary fungal infections in patients with hematological malignancies, demonstrating the efficacy and feasibility of thoracoscopic surgery in this context (16). Furthermore, there is a report of successful robotic-assisted left upper lobe resection in a patient with acute myeloid leukemia complicated by pulmonary mucormycosis, resulting in a smooth postoperative course and significant clinical improvement (17). Despite these findings, no specific studies on thoracoscopic surgery for pediatric pulmonary mucormycosis currently exist. This case provides valuable insights and methods, aiming to contribute empirical guidance for the future clinical management of similar pediatric cases.

In this case, after treatment, the inflammation caused by fungal infection in the middle and lower lobes of the right lung of the child significantly subsided, indicating that the antifungal treatment had achieved certain results. However, the inflammation in the upper lobe of the right lung remained severe and hemoptysis occurred. At this juncture, we faced a critical decision: should we continue with the previous conservative treatment or opt for surgical resection of the right upper lobe? We convened a hospital-wide multidisciplinary team (MDT) to thoroughly discuss and evaluate the feasibility and potential benefits and risks of each treatment option with the child's parents. After careful consideration, the parents opted for surgical resection of the right upper lobe. The surgery proceeded smoothly, and the postoperative recovery was uneventful. One month after the surgery, the child resumed chemotherapy, and the prognosis remains favorable.

## Conclusion

The treatment of childhood ALL complicated with pulmonary mucormycosis is particularly challenging and associated with a high mortality rate. For children with severe infections, combining antifungal therapy, such as L-AmB, with bronchoscopy and thoracoscopic surgery has been shown to be both feasible and effective.

## Data Availability

The datasets presented in this study can be found in online repositories. The names of the repository/repositories and accession number(s) can be found in the article/Supplementary Material.
