# Correction: Case Report: Fiberoptic bronchoscopy and thoracoscopic surgery as a treatment for pulmonary mucormycosis in pediatric acute lymphoblastic leukemia

**DOI:** 10.3389/fped.2025.1704333

**Published:** 2025-10-28

**Authors:** Zhenlei Jia, Yujie Qian, Lili Zhang, Yun Li, Zhiguo Chen, Ling Zhao, Juan Du, Guangxin Tuo, Fang Yue

**Affiliations:** ^1^ Department of Thoracic Surgery, Hebei Provincial Children’s Hospital, Shijiazhuang, China; ^2^ Department of Pathology, Hebei Provincial Children’s Hospital, Shijiazhuang, China

**Keywords:** fiberoptic bronchoscopy, thoracoscopic surgery, pulmonary mucormycosis, pediatric, acute lymphoblastic leukemia

A Correction on Case Report: Fiberoptic bronchoscopy and thoracoscopic surgery as a treatment for pulmonary mucormycosis in pediatric acute lymphoblastic leukemia By Jia Z, Qian Y, Zhang L, Li Y, Chen Z, Zhao L, Du J, Tuo G and Yue F (2025). Frontiers in Pediatrics. 13:1591953. doi: 10.3389/fped.2025.1591953

In the original published article, the funder Medical Science Research Project of Hebei (No. 20231149) was erroneously omitted. The full funding statement is below:

The authors declare that financial support was received for the research and/or publication of this article. Funding was received from the Medical Science Research Project of Hebei (No. 20231149).

The original version of this article has been updated.

